# Radiographic and HRCT imaging findings of chronic pulmonary schistosomiasis: review of 10 consecutive cases

**DOI:** 10.1259/bjrcr.20180088

**Published:** 2019-05-16

**Authors:** Giovanni Foti, Federico Gobbi, Andrea Angheben, Niccolò Faccioli, Carmelo Cicciò, Giovanni Carbognin, Dora Buonfrate

**Affiliations:** 1 Department of radiology, IRCCS Sacro Cuore Don Calabria Hospital, Negrar, Verona, Italy; 2 Centre for Tropical Diseases, IRCCS Sacro Cuore Don Calabria Hospital, Negrar, Verona, Italy; 3 Department of radiology, Policlinico GB Rossi, Verona, Italy

## Abstract

**Objective:**

To describe the chest radiography (CR) and the high resolution CT (HRCT) imaging findings of chronic pulmonary schistosomiasis (CPS)

**Methods and materials:**

This retrospective study included 10 patients suffering from CPS, studied between September 2013 and October 2016 by using CR and HRCT. Images were reviewed by two experienced radiologists in consensus, blinded to clinical data. A *p* value < 0.05 was considered significant

**Results:**

All the patients enrolled showed some abnormalities at HRCT, including lung consolidations, solid nodules, nodules with peri-nodular halo, ground-glass opacities, enlarged hilar lymph-nodes. Only seven patients showed findings at CR ( *p* = 0.001). At CT, none of the patients had significant pleural, vascular (pulmonary arteries) or cardiac findings. Post-therapy studies (mean interval 35 days) demonstrated the absence of residual disease in all patients.

**Conclusion:**

The imaging findings of CPS varied widely in our study population. HRCT may show signs which are occult on plain radiograph. All lesions disappeared after appropriate therapy at imaging follow-up studie.

## INTRODUCTION

Schistosomiasis is a parasitic infection common in parts of Africa, South America, the Middle East and Asia, where is a leading cause of morbidity and mortality.^1^ Acute schistosomiasis, also called Katayama syndrome, is a systemic hypersensitivity reaction against the migrating schistosomula and eggs, occurring a few weeks to months after the primary infection.^2^ Imaging features of acute infections have been described in patients with primary exposure, and are commonly seen in individuals traveling through endemic regions.^3–5^ Micro and macro-nodules, with or without perinodular ground glass attenuation areas (GGO), represents the most common findings in the acute phase at CT.^4–11^ Also ill-defined pulmonary nodules, pleural and pericardial effusion have been described on chest radiography (CR) in case reports.^12,13^

In individuals within endemic regions, chronic complications most often develop from progressive and recurrent infection. In the lungs, granuloma formation and fibrosis around the *Schistosoma* eggs retained in the pulmonary vasculature may result in an obliterative arteriolitis and pulmonary hypertension.^13^ Imaging features of chronic pulmonary schistosomiasis (CPS) have not been extensively described yet. Imaging findings can vary widely, with possible problems of differential diagnosis with other infective or non-infective diseases.^14^

This paper describes the CR and HRCT findings of a cohort of 10 patients coming from African endemic regions.

## Patients and methods

### Patient population

In this retrospective study we evaluated the imaging characteristics of patients with a diagnosis of chronic schistosomiasis admitted at the Centre for Tropical Diseases (CTD) of our hospital, between September 2015 and October 2017. Inclusion criteria consisted in the presence of at least a pulmonary lesion, with available pre and post-treatment CR and HRCT.

### Imaging technique

All patients underwent antero posterior (AP) and left lateral-lateral (LL) chest radiographs with a commercially available CR machine (Fujifilm DR velocity unit). HRCT was performed by using a commercially available CT scanner (Siemens 64 cardiac sensation, Germany), by using the following imaging parameters: slice thickness 0.75 mm, pitch 1,4, KV 120, mean mAs 90 (range 70–100, with automated current modulation); images were reconstructed at 1 mm thickness with lung window (Kernel B70f). A contrast enhanced scan was acquired in 3 of 10 patients after the intravenous administration of 1.5 ml/kg of Iopromide (Ultravist370, Bayer-Schering, Germany, 370 mg of iodine/ml) with a power injector, at a flow rate of 2 ml s^−1^ with 70 s delay. Post-processing was performed on a dedicated 3D processing workstation (Syngo MMWP VE36A, Siemens AG, Berlin, Germany). Pre-treatment CR and HRCT were performed within few days in all patients (mean 1 day; range 0–3 days).

### Image and statistical analysis

CR and HRCT images were retrospectively reviewed by two experienced radiologists in consensus (11 and 12 years of experience, respectively) blinded to clinical and pathological data. Presence of nodular opacities, solid nodules with peri-nodular halo, pulmonary consolidation, GGO, interstitial thickening, cystic lesions and pleural effusion were assessed. At HRCT hilar lymph nodes (short axial diameter >10 mm), size of main trunk of pulmonary artery (PA), of right (RPA), left pulmonary artery (LPA), and the ratio of pulmonary artery to ascending aorta (PA:AO) were also evaluated. At post-therapy exams the presence of residual disease, or the presence of pulmonary scars were assessed.

Given the small sample size and the abnormal distribution of data, nonparametric methods were used to test for correlation (Spearman’s rank correlation) or differences between groups (Kruskal-Wallis). A value of *p* < 0.05 was considered statistically significant. All statistical analyses were performed by using statistical software (SPSS Statistics Standard, ver. 18.0.0, IBM, Chicago, IL, USA).

## Results

**Figure 1.**
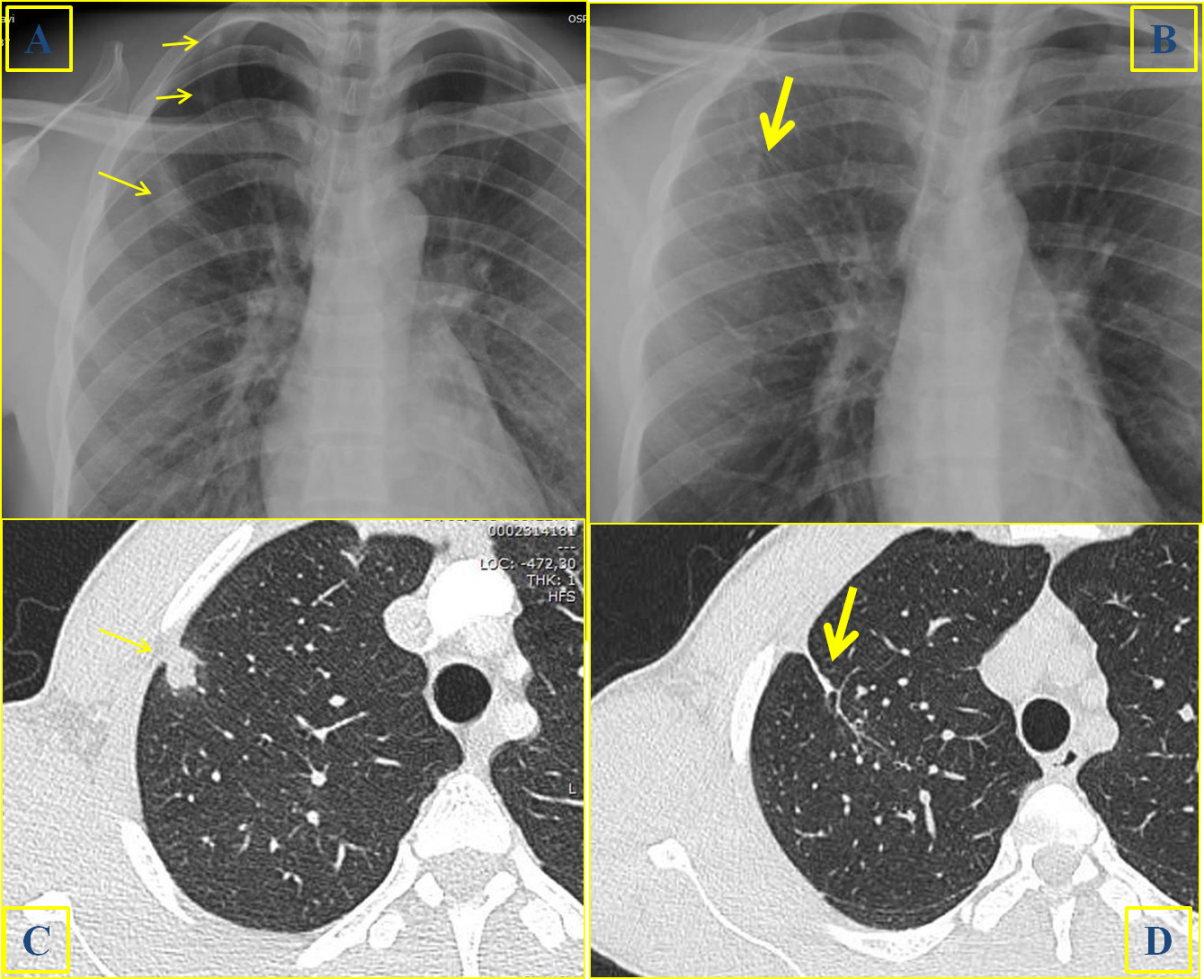
A 27-year-old male with pulmonary schistosomiasis. A prevalently solid nodule (arrow) can be identified at baseline CR (1A) and HRCT (1C); two additional nodules can be recognized in the apical region at CR (1A). The CR (1B) and HRCT (1D) scan acquired 3 weeks later, after medical treatment with praziquantel, showed the absence of any residual disease. Thin parenchymal striae (thick arrow) can be visualized, representing a parenchymal scar.

**Figure 2.**
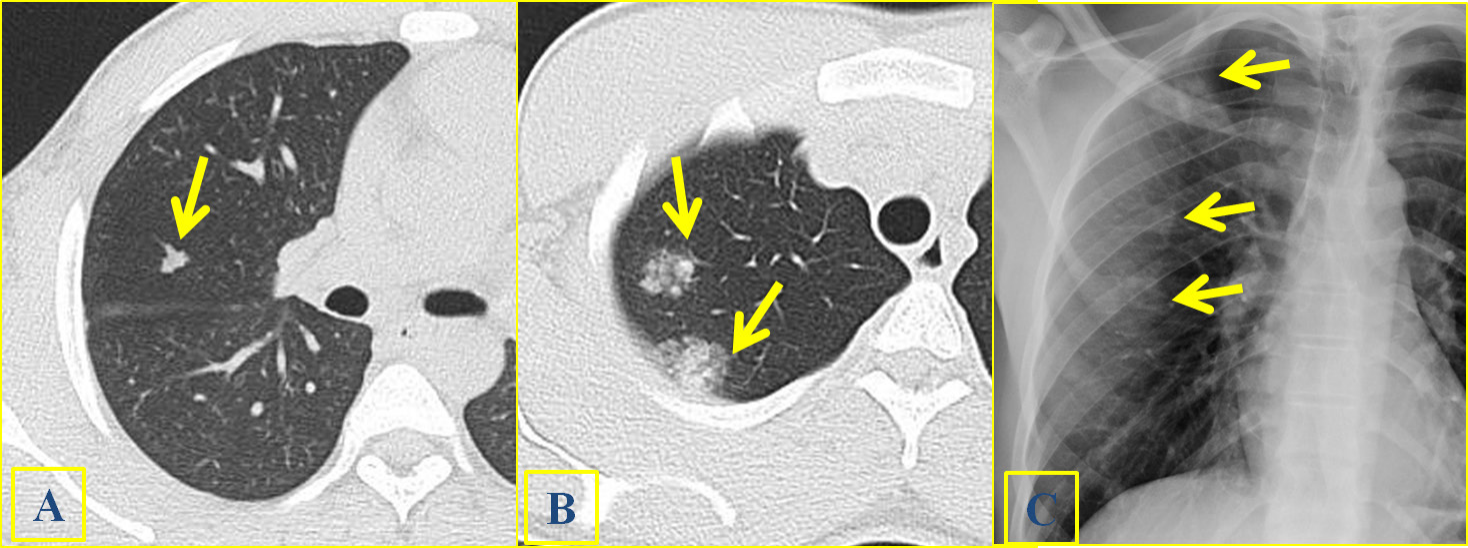
A 25-year-old male with clinical suspect of schistosomiasis and absence of pulmonary clinical findings. Multiple nodules (arrows) were identified at HRCT, including a solid lobulated nodule (2A) and two contiguous mixed nodules with peri-nodular halo on the upper lobe (2B). Solid and mixed nodules (arrows) can also be easily identified on CR (2C). The diagnosis of schistosomiasis was achieved by means of CT guided biopsy.

**Figure 3.**
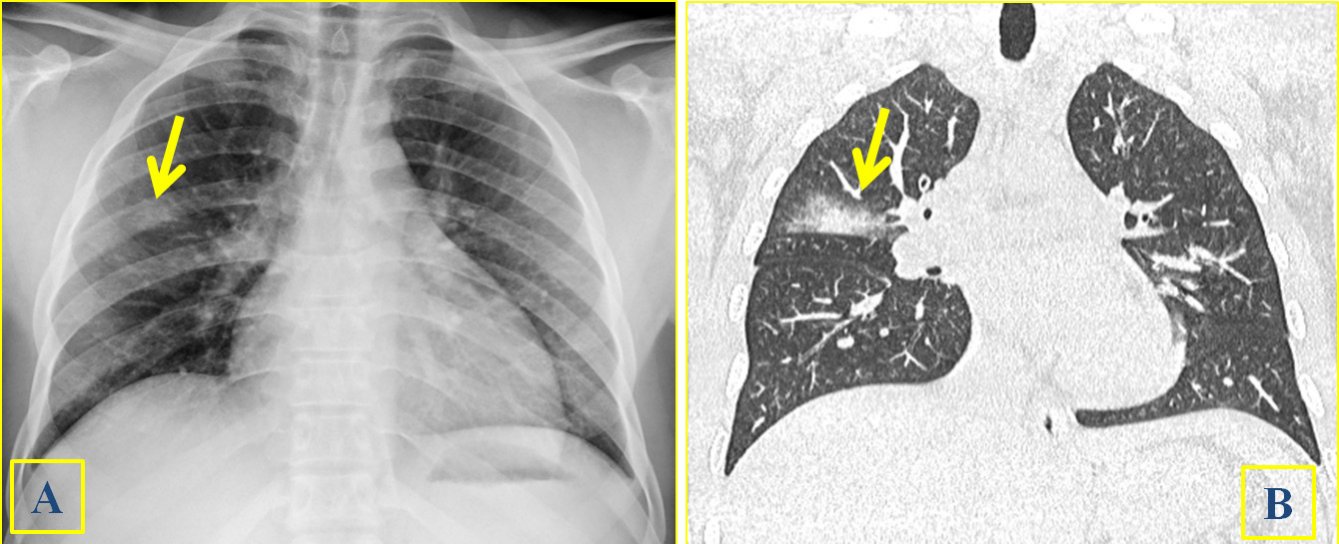
A 28-year-old male with clinical suspect of schistosomiasis (3A-3B). Lung consolidation (arrows) can be identified close to fissure at CR (3A) and at HRCT (3B).

**Figure 4.**
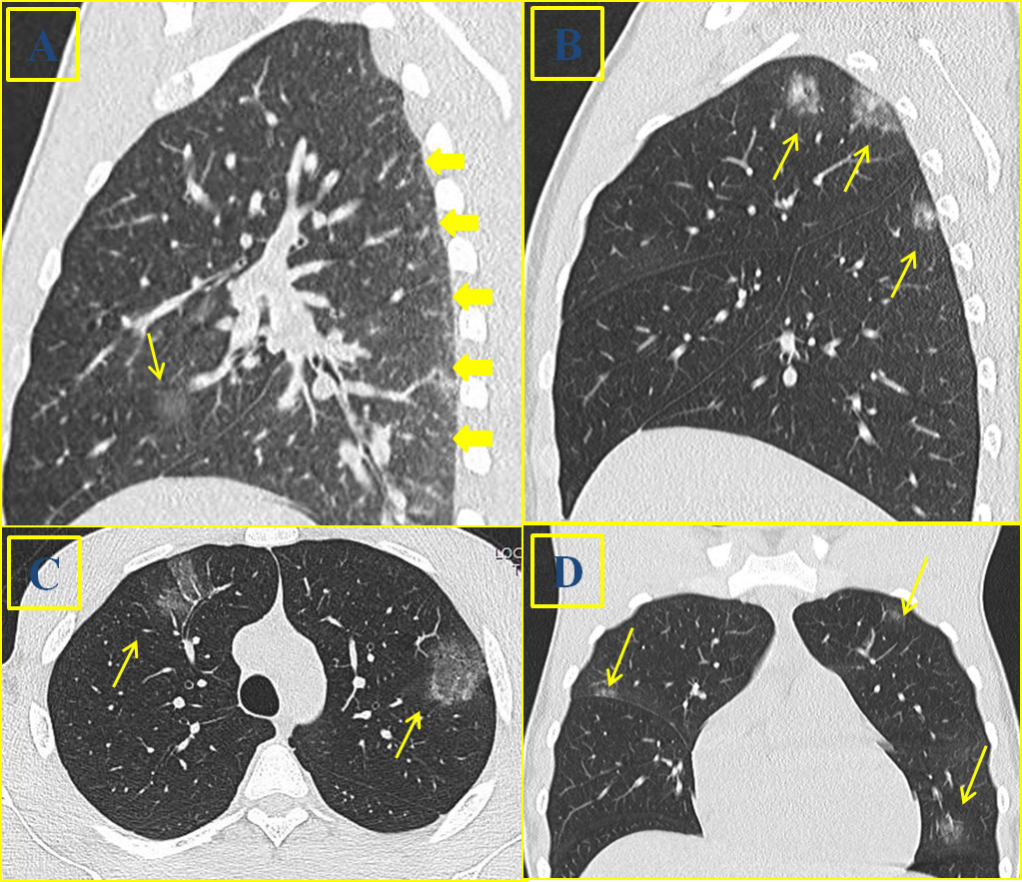
An 18-year-old male suffering from HBV hepatopathy. Multiple ground glass opacity of different size and density (arrows) can be identified close to the pleura at HRCT on sagittal (4A), axial (4C) and coronal (4D) images. Mixed nodular lesions with peri-nodular halo are detected on sagittal reconstructed image (4B)

**Figure 5.**
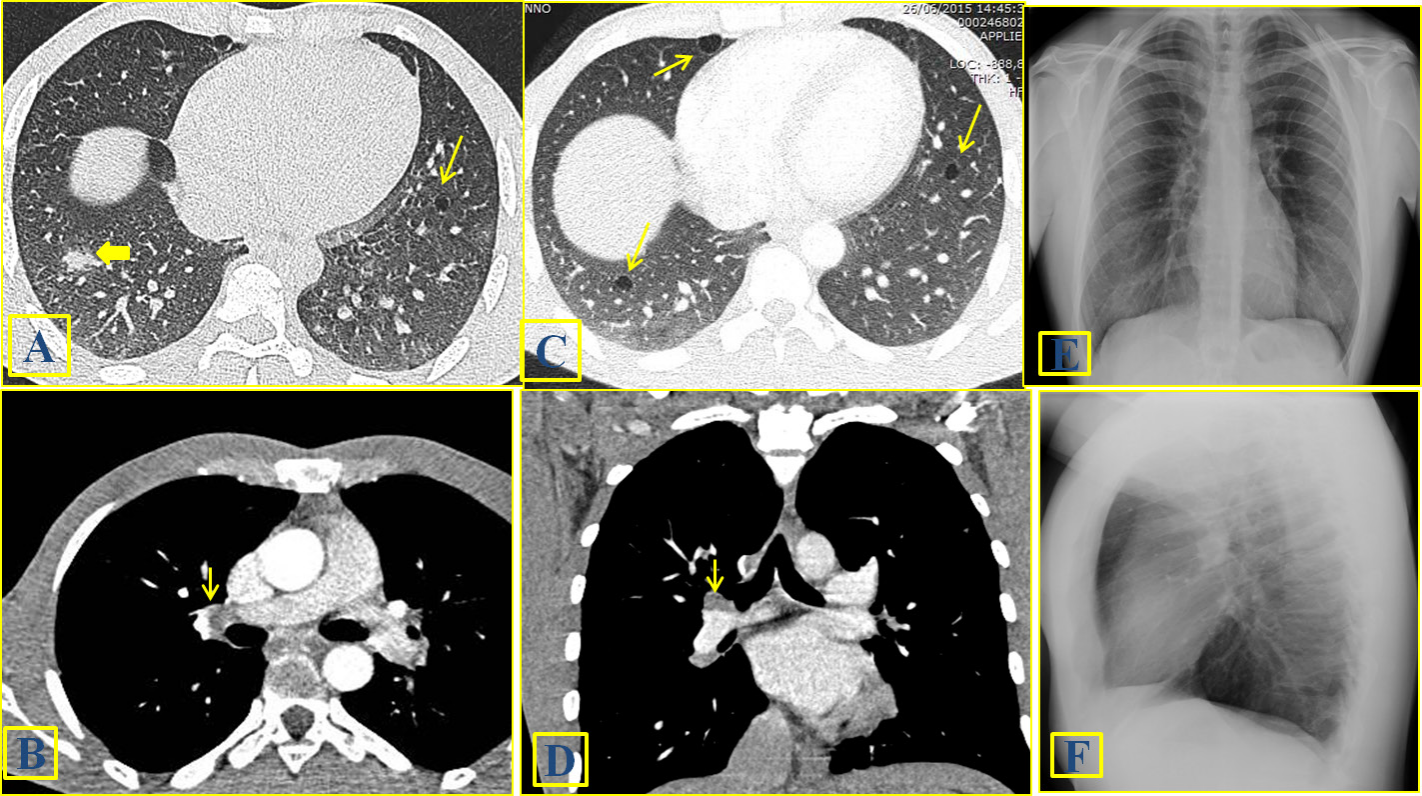
A 29-year-old male suffering from schistosomiasis and abdominal pain. Multiple tiny cystic areas (arrows) can be identified in the lower lobes (5A and 5C). A nodular area (thick arrow) can be recognized on the right side (5A). Two contiguous right hilar adenopathy (arrows) can be identified in the axial (5B) and coronal contrast-enhanced reformatted images (5D). The chest radiographs (5E, 5F) showed no abnormal findings.

Ten patients were included in our study. All patients were male immigrants born in countries endemic for schistosomiasis, and their age ranged from 18 to 35 years (mean 23.8 years). Three patients were asymptomatic and presented to the CTD for a screening program for migrants. One patient was found to have tuberculosis (PCR positivity for MTB on bronchoalveolar lavage) co-infection. In addition to schistosomiasis, one patient suffered from strongyloidiasis. The main clinical and laboratory data of patients are summarized in Table 1. Imaging findings of CR and HRCT are summarized in Table 2 and in Table 3, respectively. CT guided biopsy was performed in four cases. HRCT identified chest abnormality in all patients, whereas CR in seven patients only. Solid or mixed nodules (Figures 1 and 2) were identified at CR in five patients and at HRCT in eight patients (*p* = 0.09). A total of 15 nodules were identified at CR (Figure 1A), ranging from 1 to 6 (mean 2.5). A total of 91 nodules were detected at HRCT (Figure 1B), ranging from 1 to 28 at HRCT (mean 11.3). There was a statistically significant difference between the number of nodules detected (*p* = 0.001). Lung consolidation was detected at CR and at HRCT in the same three patients. GGO (Figures 3 and 4) were identified at CR in four patients, whereas at HRCT in eight patients. Cystic lesions (Figure 5A and C) were detected only by using HRCT in two patients. Two right hilar lymph nodes (10 mm and 12 mm, respectively) were detected at HRCT in one patient (Figure 5B and D). At CT the size of PA (mean 23 mm, range 20–25 mm), RPA (mean 16.7 mm, range 14–19 mm) and LPA (mean18.6 mm, range 14–21 mm) were within the normal range in all cases. The PA:AO ratio was within normal range (<1) in all cases (mean 0,82; range 0.78–0.93). None of the patients had significant pleural, or cardiac findings. The mean interval between the first exam and the post-therapy studies was 35 days (range, 15–92 days). In no cases there were any residual nodules or lung consolidation after therapy with praziquantel both at CR and HRCT (Figure 1B and D). At CR, a pulmonary scar was identified in two patients. At HRCT, a scar was detected in four cases.

**Table 1.** Demographics, clinical characteristics, eosinophil count, and IgE values of the patients enrolled.d

**Pz**	**Age**	**Country of origin**	**Comorbidities and/or symptoms**	**Blood Eosinophils** **Absolute count (%**)	**Blood IgE** (**UI/mL**)	**Schistosome eggs in urines/ stool**	**Serology calculated OD (cut off 0.9**)	**Lung biopsy**	**CR/HRCT** **Delay**
1	27	Mali	Pulmonary TB HBV hepatopathy Abdominal pain	750 (13·9)	4,950	*S. mans*	4.07	*S. mans* eggs	*0* *days*
2	25	Guinea	None	440 (9·6)	1,440	None	2.44	*S* spp eggs	*1* day
3	28	Ivory Coast	Abdominal pain Cough	790 (12·9)	2,930	*S. haem* *S. mans*	3.62	*S* spp eggs	*0* *days*
4	19	Senegal	Strongyloidiasis Haematuria	2040 (31)	497	*S. haem*	1.33	*S* spp eggs	*1* day
5	18	Mali	HBV hepatopathy Asymtpomatic	1960 (22)	408	*S. haem* *S. mans*	3.84	Not performed	2 days
6	21	Mali	Haematuria	250 (2·3)	2,500	*S. haem*	2.59	Not performed	*1* day
7	18	Nigeria	Cough Chest pain Abdominal pain	400 (6·6)	1,040	*S. haem*	2.11	Not performed	*1* day
8	35	Mali	HBV hepatopathy Asymtpomatic	740 (10·3)	1,370	*S. haem*	1.88	Not performed	3 days
9	18	Mali	Chest pain	290 (6·5)	77	*S. mans*	1.62	Not performed	3 days
10	29	Guinea	Abdominal pain	5,400 (55·3)	8,990	*S. haem* *S. mans*	2.74	Not performed	2 days

S. mans: S. Mansoni; S. haem: S. Haematobium; S spp: Schistosoma species

**Table 2.** CR imaging findings

Pz	NOD	CONS	GGO	LN	PLEURAL EFFUSION	POST THERAPY
1	No	No	No	No	No	No
2	1	No	No	No	No	No
3	2	No	Yes	No	No	No
4	2	No	Yes	No	No	No
5	4	Yes	Yes	No	No	Scar
6	6	Yes	No	No	No	Scar
7	No	Yes	Yes	No	No	No
8	No	No	No	No	No	No
9	No	No	No	No	No	No
10	No	No	No	No	No	No

NOD = number of nodules; CONS = consolidation; GGO = ground glass opacity; LN = lymph nodes

No = absence of imaging findings.

**Table 3.** HRCT imaging findings

Pz	NOD	CONS	GGO	LN	PLEURAL EFFUSION	CYST	PA (mm)	POST THERAPY
1	No	No	Yes	No	No	No	22.5	No
2	2	No	Yes	No	No	No	20	No
3	4	No	Yes	No	No	No	25	No
4	7	No	Yes	No	No	No	21	No
5	11	Yes	Yes	Yes	No	Yes	24	Scar
6	16	Yes	Yes	No	No	No	26	Scar
7	22	Yes	Yes	No	No	No	23	Scar
8	28	No	No	No	No	No	22.5	No
9	No	No	Yes	No	No	Yes	25	No
10	1	No	Yes	No	No	No	21	No

NOD = number of nodules; CONS = consolidation; GGO = ground glass opacity; LN = lymph nodes; PA = size of pulmonary artery (in mm). No = absence of imaging findings.

## Discussion

With increasing migration flow, the diagnosis of chronic schistosomiasis in Western countries is becoming more and more frequent.^15^ This is due to the fact that, in endemic areas, the first Schistosoma infection is invariably acquired in childhood, acute infections being invariably diagnosed in travelers.^1^ Imaging features in the radiological literature have been mostly described in case reports or small case series.^16–19^ Imaging findings in our study included micro and macro-nodules with and without halo, GGO areas alone, lung consolidation, hilar adenopathy, thickening of interlobular septa, cystic areas. The nodules ranging from 5 to 20 mm were mainly well defined, whereas large nodules (>20 mm) were mainly ill-defined due to the presence of peri-nodular halo. There was no predominance as regards the position of imaging findings in the lung base or apex. Also there was no central or peripheral predominance.

All patients of our series presented with at least an abnormality at HRCT. Lung lesions ranged from the presence of a single GGO area to the presence of diffuse lung disease, including interstitial and alveolar burden. CR clearly showed the evidence of chest disease in seven of the 10 patients. However, small areas of GGO alone, micro-nodules measuring 5 mm or less, but also medium size nodule with prevalent peripheral halo, may be missed at chest CR. As previously reported, HRCT represents the reference imaging tool for the diagnosis of subtle or atypical interstitial findings. For this reason we believe that HRCT should be performed in all patients with clinical suspect of chronic schistosomiasis at first assessment, despite absence of findings at CR. In our series, the patients with normal CR showed only subtle imaging findings at HRCT. One patient had a single GGO focus in the sub pleural region of inferior right lobe, one patient had four micro-nodules, and a patient had three small nodules with prevalent peri-nodular GGO halo. Even if subtle, these findings may represent the key for diagnosis in difficult cases.

Also, the involvement of the lungs in the chronic phase was described in relation to pulmonary hypertension.^20^ In the study of Hoette et al pulmonary artery enlargement was more pronounced in schistosomiasis-associated pulmonary arterial hypertension than in idiopathic pulmonary arterial hypertension.^20^ We had no cases of PA, RPA and LPA enlargement. Possible explanation is the different age of patients enrolled, with a mean age of 48 years in the study by Hoette et al, versus a mean age of 23,8 years in our study. Also it must be underlined that, since the patients enrolled were mainly asymptomatic, we did not evaluate pulmonary pressure. Long term follow-up studies could be useful to understand if any progressive enlargement of PA may occur lately.

There was no pleural or pericardial effusion in our series. Chronic inflammation around the migrated eggs within lung, in peri-arteriolar regions, could be an explanation for pleural sparing in this phase.

Lung biopsy was performed only in four patients to confirm the diagnosis. in the remaining biopsy was avoided and the diagnosis was confirmed once lesions disappeared following appropriate therapy.

All the patients enrolled were indeed successfully treated with praziquantel. The optimal timing for the follow-up study after therapy could be considered between 2 and 4 weeks (mean delay of 35 days in our study). This relatively short time could be helpful not only for the optimization of patients management, but above all to exclude other possible causes of lung nodules. The possibility to mimicking other infective and not-infective disease have been described in the literature for lung schistosomiasis.^17–19^ Especially in case of single lesions, or in the presence of an index lesions, biopsy could be considered for diagnosis.^15^ The disappearance of imaging findings after therapy could therefore be important to achieve diagnosis, avoiding useless biopsy. Although less accurate for identifying subtle pulmonary lesions, CR can be very useful for monitoring response to therapy during follow-up. CR correctly showed the absence of residual disease or the presence of a pulmonary scar in all patients presenting lung consolidation. Therefore, a reduction of radiation exposure and a reduction of costs could be achieved, obviating the need of repeating HRCT in these cases.

## Conclusion

Chronic pulmonary schistosomiasis imaging findings may vary widely and may not correlate with symptoms. HRCT could show subtle imaging findings which are missed on plain radiograph. CR may represent a cheap and widely available imaging tool for demonstrating the absence of residual disease after appropriate medical treatment.

## Learning points

- Solid or mixed nodules represented the most frequent imaging findings of CPS in our series.
- HRCT may show subtle imaging signs which are occult on plain radiographs.
- All the lesions in the lungs disappeared after appropriate medical treatment. In case of clinical suspect, this issue may represent a key for differential diagnosis, avoiding the need of any additional invasive procedures.
